# The Multiple Avenues of Contribution of Robert McMahon to Prevention Science

**DOI:** 10.1007/s11121-025-01809-8

**Published:** 2025-04-21

**Authors:** Patrick H. Tolan

**Affiliations:** University of Virginia, Charlottesville, VA USA

**Keywords:** Preventin, Developmental psychopatholgy

## Abstract

This issue of *Prevention Science*, focused on studies influenced by and reflective of the contributions of Robert McMahon, contains a diverse set of studies focused across multiple important areas of our field. This commentary focuses on that breadth and diversity of contributions as the core of McMahon’s role in prevention science. Several specific examples of such impact integrating knowledge across clinical child psychology, developmental psychopathology, and prevention studies are described, and the linkages to articles in this volume noted. Also, noteworthy is the extension of McMahon’s influence through collegial and collaborative efforts by convening and facilitating scientific exchange. He has provided a connective enhancement to our field that has benefitted many and continues to influence towards excellence.

This issue of *Prevention Science*, focused on studies influenced by and reflective of the contributions of Robert McMahon, contains a diverse set of studies addressing multiple important issues in our field. In fact, it may be the diversity and breadth of topics that is the most remarkable feature, particularly for relating these works to McMahon’s role. He has provided essential theoretical formulations and empirical contributions to our body of knowledge in several areas, which have been subsequently incorporated into the conduct of studies by colleagues and students. Moreover, through numerous collaborations and facilitative roles such as editor of *Prevention Science*, director of a major violence prevention center, and convener of the Banff International Conferences on Behavioural Science (2025) for more than 40 years, he has improved the extent and efficiency of knowledge sharing and cross-fertilization of approaches. Thus, it is apropos that one summarization of this volume is that the diversity of topics reflects the extent to which McMahon has been involved with and served as a distinguished connector of distinct topics of inquiry.

A corollary notion is that he not only has functioned as an influence in prevention science but has also integrated this field with methods and approaches emanating from child clinical psychology and developmental psychopathology (Dishion & McMahon, 1998). McMahon is the rare scholar who, rather than work in one field, has articulated the complimentary roles of these ways of conceptualizing child prevention issues and was a leader in the integration of such perspectives. His work stands as a model of thoughtful, systematic, complex integration for theory advancements, building effective prevention programming, and engendering practices and policy implications that translate to plausible large-scale impact. Examples of areas of primary influence can be found among the preventive intervention focused studies within this Special Issue.

Multiple articles include an emphasis on intervention process and component determination informed by developmental appropriateness (Craig et al., 2025; Heilman et al., 2025). Similarly, several articles focus on interventions based on prior tested components, procedures, and delivery methods (Craig et al., 2025; Prinz et al., 2024; Stormshak et al., 2024). One of McMahon’s early and most influential contributions was *Helping the Noncompliant Child* (Forehand & McMahon, 1981; McMahon & Forehand, 2003). That volume set a model for the precise formulation of intervention targets and chosen methods based on systematic empirical study incorporating developmental considerations. Over the five plus decades since that book first appeared, the growth of empirical tests has led to the ability to not only test specific interventions but also to apply meta-analytic approaches across intervention tests (Canário et al., 2024; Craig et al., 2025) and explore differential responses among intervention recipients (Heilman et al., 2025; McCabe et al., 2024). Among the prevention intervention studies in this volume, there are foci on efficacious procedures, practical organization and delivery, developmental sensitivity, attention to differential impact, tracking impact on long-term outcomes, and building knowledge through programmatic studies, all of which reflect direct contributions of and influence derived from studies by McMahon and colleagues. Further, in the two articles in this volume reporting meta-analyses of behavioral family interventions (Canário et al., 2024; Craig et al., 2025), the influence of McMahon’s work can be seen in the conceptual frames applied in grouping of studies and what questions are addressed with the analyses. For example, each focuses on more than their overall benefit, examining mechanisms of impact and variation in effect, topics McMahon has been a proponent of studying to improve family-based prevention.

Prevention programs have advanced profoundly in significance since the initial work in the early 1990s, with McMahon and his colleagues within the Fast Track group (i.e., Conduct Problems Prevention Research Group; e.g., Dodge et al., 2015) were among those first exploring efficacy through a developmental lens and randomized controlled trials. The thoughtful, complex, and impeccable organization and execution of the Fast Track project meant that McMahon and his colleagues often were at the forefront of analytic method applications, ability to address emerging questions, and discovery of complexities and nuances previously unrecognized. This is still the case. In this Special Issue, the Fast Track group provides new analyses linking early impact of the intervention on later shifts in risk, tracing that long-term path impact indirectly as well as directly (McCabe et al., 2024).

A different form of influence of McMahon is through work as a colleague and co-explorer with others with their own concurrently developing prevention approaches. One example is of the report on contextual conditions in understanding impact with the Family Check-Up prevention program (Bennett et al., 2024). Another example is the article by Turner and Sanders (2024), which reviews the Triple P-Positive Parenting Program. As the authors explicitly noted, McMahon was a peer supporter of their work. As they explain how the approach was formulated, elaborated, and expanded over time, one can see an approach also found in a review of McMahon’s publications; systematic interplay between clinical child psychology, developmental psychopathology, and prevention science with each advance tested empirically and modified in formulation as needed based on experimental testing. McMahon’s impact on the field indirectly has been through generosity as a colleague, helping many peer scientists to improve their work and advance through careful formulations and increasingly elaborate formulations of variations in application of a given central approach (Suarez et al., 2024). This has been my experience throughout our collegial relationship.

My own collegial benefit from McMahon started from the moment I first met McMahn. Our Metropolitan Area Child Study and the Fast Track project were both initially funded in 1990 as a NIMH initiative with a fundamental purpose of conducting randomized controlled trials to test whether youth violence could be prevented. Joint meetings were set up to link coordination and exchange ideas. As our roles in our respective projects included leading on the family intervention aspect of the program, McMahon and I were paired up for specific discussions and often had similar topic responsibilities in overall group deliberations. My initial impression, and one borne out over subsequent decades, was that his understanding is deep and broad, with resultant carefully and soundly formulated perspectives. Problem solving and exchange of ideas are based in experience along with full command of the pertinent research, applying it dispassionately to his own work as well as others. The clear priority was to promote the best work. In addition to our shared interest, I gained help from him immediately. For example, while he was adapting a clinical child intervention with previous empirical support, our family group approach had only been piloted, being derived from extending family therapy for adolescent delinquency (Tolan et al., 1986). Subsequent regular generous consultation and collegial engagement have continued. Shared findings, numerous meetings, and ancillary conversations over the ensuing decades have certainly enriched my thinking and learning overall, with frequent specific improvements in the work due to his acumen. I, like many others, have benefitted in multiple ways via the broad and varied contributions of McMahon to our field. It is a pleasure to witness the abundant evidence of his impact and to contribute due appreciation.
